# Case Report: A case of recurrent thrombosis in pediatric antiphospholipid syndrome associated with pediatric onset systemic lupus

**DOI:** 10.3389/fped.2022.1004053

**Published:** 2023-02-03

**Authors:** Lingjuan Liu, Liqun Liu, Lu Zhang, Peng Huang, Xiqiang Dang, Lanjun Shuai, Xingfang Li, Yongzhen Li, Dingan Mao, Xiaochuan Wu, Yan Cao

**Affiliations:** ^1^ Department of Pediatrics, The Second Xiangya Hospital of Central South University, Changsha, China; ^2^ Department of Pediatric Neurology, patientren's Medical Center, The Second Xiangya Hospital of Central South University, Changsha, China; ^3^ Department of Pediatric Nephrology, patientren's Medical Center, The Second Xiangya Hospital of Central South University, Changsha, China

**Keywords:** systemic lupus erythematosus, nephrotic syndrome, antiphospholipid syndrome, antiphospholipid antibody, gross hematuria

## Abstract

Systemic lupus erythematosus (SLE) is an autoimmune disease with multi-system involvement as the main manifestation, and has complex and diverse clinical features. Studies on large samples have revealed that SLE patients have a significantly increased risk of thrombotic events, which are also one of the important causes of morbidity and mortality in SLE patients. Antiphospholipid syndrome (APS) is a rare autoimmune disorder characterized by recurrent arterial and venous thrombosis, pregnancy-related complications, and the persistence of antiphospholipid antibodies at a 12-week interval. There are few reports about SLE coexisting with APS in children. This paper reported a school-age patient who started the disease with gross hematuria after bumping into the waist. The initial diagnosis of renal contusion was then confirmed by color Doppler ultrasound as renal vein and inferior vena cava embolism. She suddenly developed severe chest pain and dyspnea 3 days after hospitalization. And imaging supported pulmonary embolism with massive proteinuria, hypoalbuminemia, and hypercholesterolemia. The initial diagnosis was nephrotic syndrome (NS) with arteriovenous embolization, and popliteal vein embolism occurred again 5 years later, and she was thus diagnosed with SLE coexisting with APS. Afterwards, we discussed the possible mechanism and therapeutic strategies of SLE&APS that started with nephrotic syndrome, in order to achieve early identification and treatment of the disease and improve the prognosis of children.

## Introduction

Systemic lupus erythematosus (SLE) is an autoimmune-mediated diffuse connective tissue disease characterized by immune inflammation. Its main feature is the presence of multiple autoantibodies represented by antinuclear antibodies in the patient's serum and multi-system involvement (1, 2). 10%–20% of all SLE patients had the onset in childhood (3). SLE is a common cause of secondary nephrotic syndrome (NS), some SLE children started the disease with NS, and up to 2/3 of pediatric SLE patients evaluated in large medical centers had some degree of renal involvement (4, 5). APS is an autoimmune disease involving multi-systems characterized by recurrent arterial and/or venous thrombosis, abnormal pregnancy, and persistence of antiphospholipid antibodies (6). APS is classified into primary APS and secondary APS, usually associated with SLE (7). Antiphospholipid antibodies are heterogeneous antibodies acting on phospholipids and/or phospholipid-binding proteins, and are prone to cause hypercoagulability and many clinical manifestations, mainly including anticardiolipin antibodies, anti-*β*2-glycoprotein I (anti-*β*2GP I) antibodies, and lupus anticoagulants (LAC) (8, 9). The clinical manifestations related to the presence of aPL antibodies are similar in children and adults, with a predominance of venous thromboses (10).

At present, there are many reports about SLE with APS in adult patients, but few reports in children. This paper reported a school-age patient with the onset of gross hematuria after impact on the waist, who was initially diagnosed with embolic-onset NS, and was diagnosed with SLE and APS after 5 years of disease course. Moreover, the discussion based on literature was performed to strengthen the understanding of this disease.

## Case presentation

**Figure 1:**
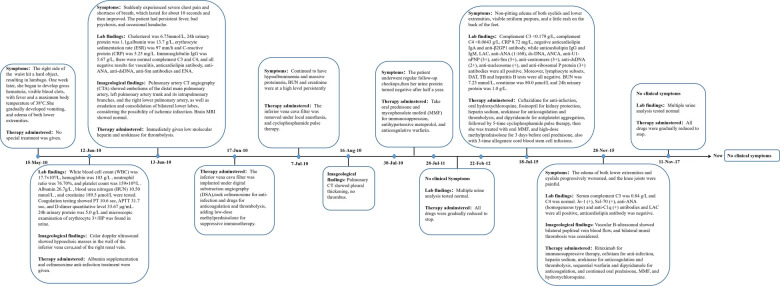
Timeline of events.

**Figure 2:**
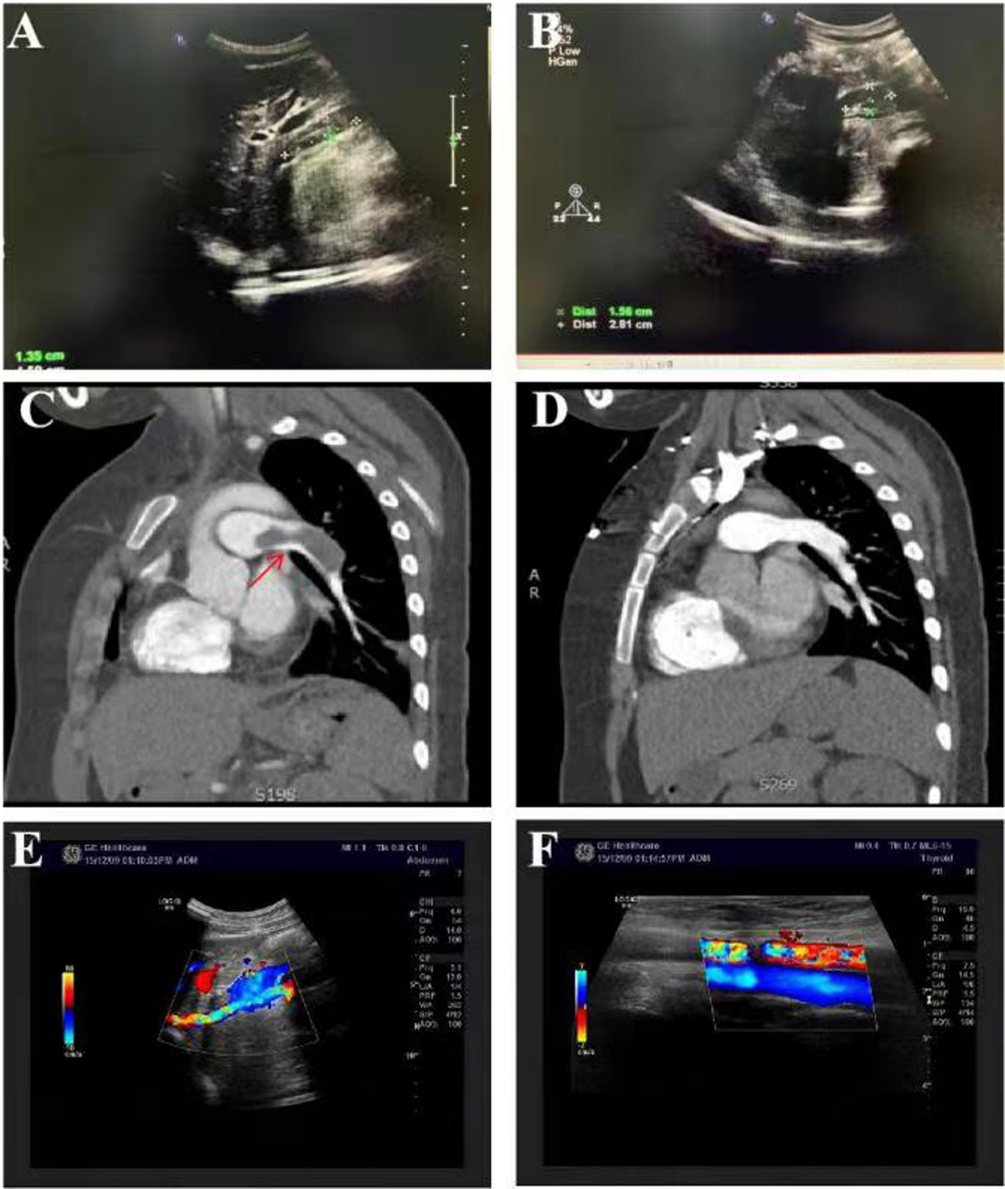
*Clinical features of the current patient.* Among auxiliary examinations, color Doppler ultrasound showed hypoechoic masses in the wall of the inferior vena cava (**A**), and of the right renal vein (**B**), considering the possibility of thrombosis. Pulmonary artery CT angiography (CTA) showed embolisms of the distal main pulmonary artery, left pulmonary artery trunk and its intrapulmonary branches, and the right lower pulmonary artery (**C**), considering the possibility of ischemic infarction. The thrombi disappeared after effective treatment (**D**). Vascular B-ultrasound showed bilateral popliteal vein blood flow, and bilateral mural thrombosis was considered (**E-F**).

**Figure 3:**
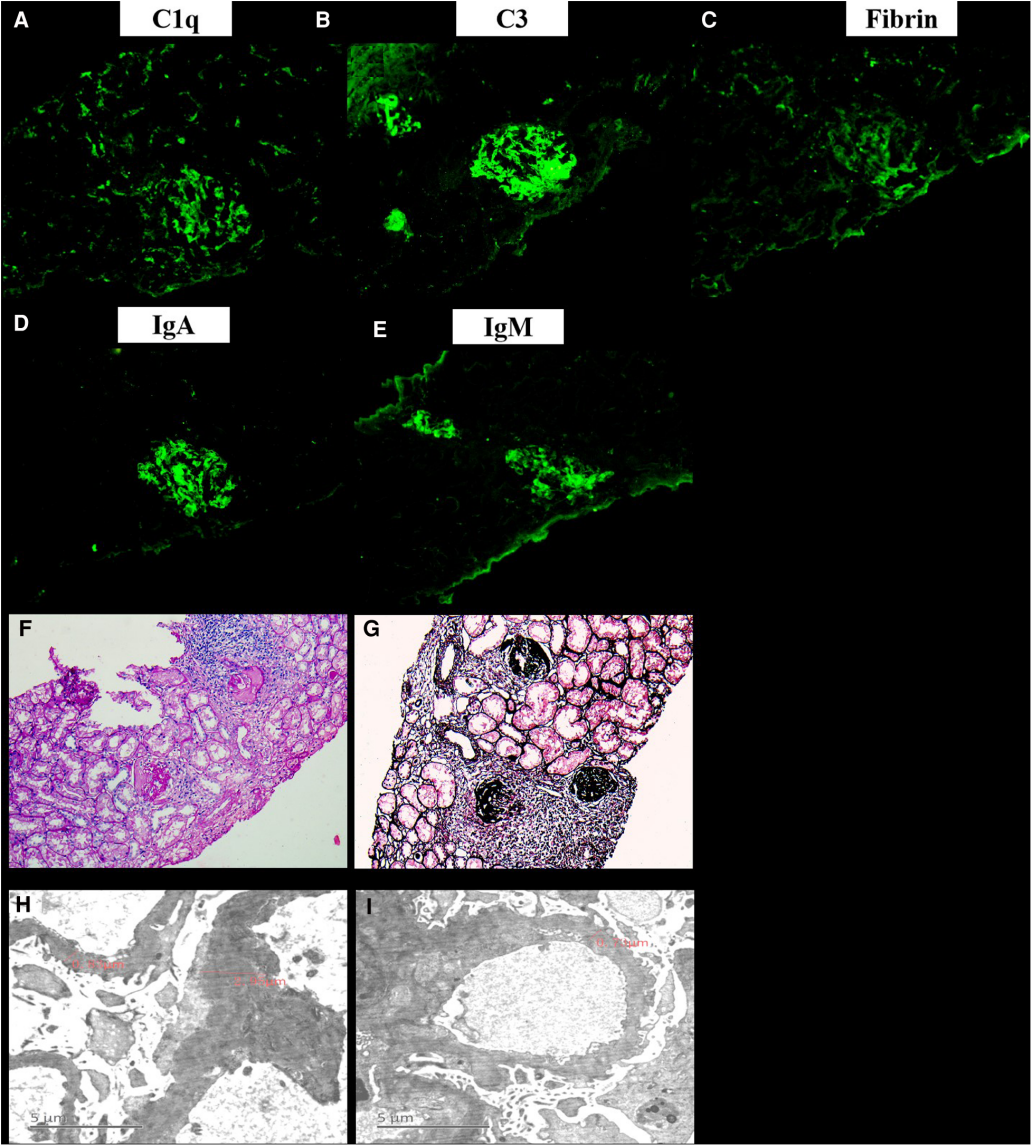
*The renal pathology of the current patient.* Immunofluorescence showed that IgA, IgG, C3, IgM, Fibrin, C4, C1q, MAB*α*-1, MAB*α*-3, and MAB*α*-5 were all positive (**A-E**). Under the light microscope, there were 11 glomeruli, 9/11 glomeruli had bulbous sclerosis, the other 2 glomeruli had increased lobulation, mesangial cells (2–4 area) hyperplasia and mesangial matrix, capillaries did not open well, and endothelial proliferation and glomerulus exudation with peribulbar fibrosis and inflammatory cell infiltration were observed. Segmental thickening of the glomerular basement membrane was found, and deposition of polyerythrophilin was observed subcutaneously in the mesangial area. Additionally, there were vacuolar degeneration of renal tubules, abundant protein tubules, and marked edema and fibrosis of renal interstitium with focal inflammatory cell infiltration (**F-G**). The mesangial cells in 2 glomeruli showed mild to moderate proliferation of matrix with abundant electron deposits. Capillary basement membrane thickness extensively thickened (800–3000 nm). There were large high-density electron deposits on the endothelial side, extensive fusion of microvilli, and partial renal tubular epithelial edema in the foot process (**H-I**).

A 13-year-old female patient was admitted to our hospital for the first time on June 12, 2010 due to bruises on the right waist for more than 20 days, gross hematuria, and fever for half a month. Figure 1 is a timeline of the patient's clinical course and treatments. The right side of the patient's waist hit a hard object, resulting in lumbago on May 15, 2010. One week later, she began to develop gross hematuria, visible blood clots, no frequent and urgent urination, and no dysuria, with fever and a maximum body temperature of 38 °C. She gradually developed nausea, vomiting, and edema of both lower extremities. Physical examination revealed normal blood pressure, normal development, pale complexion, clear consciousness, no jaundice, and no palpable enlargement of superficial lymph nodes. There was no congestion in the pharynx and no swelling of the tonsils. Cardiopulmonary examination showed no abnormalities. The abdomen was flat and soft, no abdominal mass was palpated, the liver was impalpable below the costal margin, there was no percussion pain in the bilateral kidney area, and no tenderness and rebound tenderness in the bilateral ureteral area. Pitting edema occurred in both lower limbs, muscle strength and muscle tone of the limbs were normal, and physiological reflexes were present. Color doppler ultrasound showed hypoechoic masses in the wall of the inferior vena cava (Figure 2A), and of the right renal vein (Figure 2B), considering the possibility of thrombosis. White blood cell count (WBC) was 17.7 × 10^9^/L, hemoglobin was 103 g/L, neutrophil ratio was 76.70%, and platelet count was 159 × 10^9^/L, all normal values of specific detection indexes were showed in Table 1. Albumin 26.7 g/L, blood urea nitrogen (BUN) 10.50 mmol/L, and creatinine 189.5 *μ*mol/L were tested. Coagulation testing showed prothrombin time (PT) 10.6 s, activated partial thromboplastin time (APTT) 31.7 s, and D-dimer quantitative level 33.67 *μ*g/ml. Microscopic examination of erythrocyte 3+/HP was found in urine and 24 hour urinary protein quantity was 5.0 g/L. After hospitalization, albumin supplementation and cefmenoxime anti-infection treatment were given. The day after admission, she suddenly experienced severe chest pain and shortness of breath, which lasted for about 10 s and then improved. The subsequent physical examination showed blood pressure 120-145/75-90 mmHg, transcutaneous oxygen saturation 80%, clear breath sounds in the left lung, decreased breath sounds in the right lower lung, and no dry/wet rales heard. The patient had persistent fever, bad psychosis, and occasional headache. Pulmonary artery CT angiography (CTA) showed embolisms of the distal main pulmonary artery, left pulmonary artery trunk and its intrapulmonary branches, and the right lower pulmonary artery (Figure 2C), as well as exudation and consolidation of bilateral lower lobes, considering the possibility of ischemic infarction. Brain MRI showed normal. Cholesterol was 6.75 mmol/L, 24 hour urinary protein quantity was 1.1 g, albumin was 13.7 g/L, erythrocyte sedimentation rate (ESR) was 97 mm/h and C-reactive protein (CRP) was 5.25 mg/L. Immunoglobulin IgG was 5.67 g/L, there were normal complement C3 and C4 (1.04 g/L and 0.13 g/L respectively), and all negative results for vasculitis, anticardiolipin antibody, anti-ANA, anti-dsDNA, anti-Sm antibodies and extractable Nuclear Antigen (ENA). She was immediately given low molecular heparin and urokinase for thrombolysis. The inferior vena cava filter was implanted under digital substraction angiography (DSA) on June 17, 2010. The patient was diagnosed with NS and thrombogenesis. Due to the use of anticoagulants, the patient was concerned about the risk of bleeding and the kidney biopsy was not completed. Then the patient took cefmenoxime for anti-infection and drugs for anticoagulation and thrombolysis, adding low-dose methylprednisolone for suppressive immunotherapy. However, the patient continued to have hypoalbuminemia and massive proteinuria, BUN and creatinine were at a high level persistently. The inferior vena cava filter was removed under local anesthesia, and cyclophosphamide pulse therapy was added 20 days later. On July 20, she was switched to warfarin for anticoagulation. The edema basically subsided, and the proteinuria gradually decreased. After discharge, the patient underwent regular follow-up checkups, the thrombi disappeared (Figure 2D). Then her urine protein turned negative after half a year, and she continued to take oral prednisone and mycophenolate mofetil (MMF) for immunosuppression, antihypertensive metoprolol, and anticoagulative warfarin for 1 year. As multiple urine analysis tested normal, all drugs were gradually reduced to stop on February 22, 2012. On July 18, 2015, the patient had no obvious cause for non-pitting edema of both eyelids and lower extremities, visible retiform purpura, and a little rash on the back of the feet. Laboratory inspections showed complement C3 < 0.179 g/L, complement C4 < 0.0643 g/L, CRP 0.72 mg/L, negative anticardiolipin IgA and anti-*β*2GP1 antibody, while anticardiolipin IgG and IgM, lactic acid (LAC), anti-Anti-nuclear antibody (ANA) (1:160), ds-DNA, anti-neutrophil cytoplasmic antibodies (ANCA), anti-U1-nPNP (3+), anti-Sm (3+), anti-centromere (3+), anti-dsDNA (2+), anti-nucleosome (+), and anti-ribosomal *P* protein (3+) antibodies were all positive. Moreover, lymphocyte subsets, direct antiglobulin test (DAT), tuberculosis (TB) and hepatitis B tests were all negative. BUN was 7.23 mmol/L, creatinine was 80.0 *μ*mol/L and 24 h urinary protein was 1.0 g/L. Renal pathology suggested lupus nephritis (LN) (type VI according to ISN/RPS Classification Standard) (Figures 3A–I). The patient was given ceftazidime for anti-infection, oral hydroxychloroquine, fosinopril for kidney protection, heparin sodium, urokinase for anticoagulation and thrombolysis, and dipyridamole for antiplatelet aggregation, followed by 5-time cyclophosphamide pulse therapy, then she was treated with oral MMF, and high-dose methylprednisolone for 3 days before oral prednisone. The patient underwent 3-time allogeneic cord blood stem cell infusions and was discharged from the hospital after her condition improved. The patient took medicine regularly post-discharge. The edema of both lower extremities and eyelids progressively worsened, and the knee joints were painful on November 28, 2015. Serum complement C3 was 0.84 g/L and C4 was normal. Jo-1 (+), Scl-70 (+), anti-ANA (homogeneous type) and anti-C1q (+) antibodies and LAC were all positive, anticardiolipin antibody was negative. Vascular B-ultrasound showed bilateral popliteal vein blood flow, and bilateral mural thrombosis was considered (Figures 1E,F). The patient was treated with rituximab for immunosuppressive therapy, cefotiam for anti-infection, heparin sodium, urokinase for anticoagulation and thrombolysis, sequential warfarin and dipyridamole for anticoagulation, and continued oral prednisone, MMF, and hydroxychloroquine. After discharge, the patient was regularly followed up, and the urine protein gradually turned negative, and then all the drugs were gradually stopped. Currently, she has been off medication for 5 years and is generally in good condition.

**Table 1:** The patient's results and normal values of specific detection indexes.

Detection indexs	The patient's detection results	Normal values
WBC	17.7 × 10^9^/L	4.3–11.3 × 10^9^/L
Hemoglobin	103 g/L	114–154 g/L
Neutrophil ratio	76.70%	31%–70%
Platelet count	159 × 10^9^/L	150–407 × 10^9^/L
Albumin	26.7 g/L	42–56 g/L
Blood urea nitrogen	10.50 mmol/L	2.5–6.5 mmol/L
Creatinine	189.5 *μ*mol/L	33–75 *μ*mol/L
Prothrombin time	10.6 s	10–14 s
Activated partial thromboplastin time	31.7 s	28–45 s
D-dimer quantitative level	33.67 *μ*g/ml	0–0.55 *μ*g/ml
Cholesterol	6.75 mmol/L	2.9–5.2 mmol/L
Erythrocyte sedimentation rate	97 mm/h	0–20 mm/h
C-reactive protein	5.25 mg/L	0–3 mg/L
Immunoglobulin IgG	5.67 g/L	8.6–17.4 g/L
Complement C3	1.04 g/L (13-Jun-10)	0.7–1.4 g/L
	<0.179 g/L (18-Jul-15)	
Complement C4	0.13 g/L (13-Jun-10)	0.1–0.4 g/L
	<0.0643 g/L (18-Jul-15)	

## Discussion

In the present study, we report a patient with onset at age 12 of gross hematuria, blood clots and low back pain due to bumping into the waist, followed by dyspnea and severe chest pain. Imageological examination showed embolisms in the inferior vena cava and right renal vein, pulmonary artery branches, and right lower pulmonary artery. The patient also had massive proteinuria, hypoalbuminemia, pitting edema and hyperlipidemia, and was diagnosed with embolic-onset NS. After active treatment, the clinical symptoms significantly improved. 5 years later, edema and pain in both knee joints recurred, multiple autoantibodies were positive, popliteal vein embolism was present, and combined with renal pathology, the final diagnosis was SLE coexisting with APS. The current patient had a young-onset age, thrombotic events in multiple sites, and multiple autoantibodies changed from negative to positive. Fortunately, she responded well to immunosuppressive therapy, with a good prognosis, and a high quality of life after a 5-year follow-up.

NS is caused by kidney disease with increased permeability of the glomerular filtration barrier, and embolism is the most common complication, the mechanism might result from a combination of endothelial cell damage, imbalance of the coagulation and anticoagulation system, and decreased activity of the fibrinolytic system (11, 12). Thromboembolism could usually be divided into venous thrombosis and arterial thrombosis. Although the symptoms of arterial thrombosis are more obvious than those of patients with venous thrombosis, the condition of arterial thrombosis is generally more serious, and the delayed treatment also affects the prognosis and quality of life of patients (13). Most NS patients with pulmonary vascular thrombosis do not present the classic triad of pulmonary embolism, and their clinical manifestations could range from asymptom to tachypnea and tachycardia, and even sudden death (14, 15). However, most pulmonary embolisms are occult, and studies have shown that only 1/3 of patients are symptomatic, which makes some patients vulnerable to missed diagnoses (16). The current patient was initially considered diagnosed with renal contusion due to the onset of gross hematuria after bumping into the waist, with visible blood clots, lumbago, edema in both lower extremities and fever. Angiography showed thrombi in the right renal vein and inferior vena cava. Albumin was not lower than 25 g/L despite proteinuria, which was not consistent with the diagnosis of NS temporarily. One day later, the patient had sudden severe chest pain and dyspnea, and CT showed extensive pulmonary embolism. At this time, she also developed massive proteinuria, hypoalbuminemia, and hypercholesterolemia, and the edema of both lower extremities was aggravated, which led to the diagnosis of nephrotic syndrome. Inconsistent with the previous cases, this patient had embolism as the first symptom, and both arteries and veins were involved. In order to further investigate the secondary factors, the tests for lupus, immunity, complement, antiphospholipid antibody, hepatitis and other related were completed, all of which were negative. Fortunately, the patient's symptoms were significantly relieved after effective thrombolysis, anticoagulation and immunosuppression (glucocorticoids, cyclophosphamide and MMF). Proteinuria disappeared after half a year of treatment, all drugs were gradually discontinued.

However, after 3 years of drug withdrawal, the patient reappeared with edema of the eyelids and lower extremities, accompanied by pain in the knee joints. The re-examination showed positive anticardiolipin antibody, hypocomplementemia, positive multiple autoantibodies (including anti-ANA, anti-dsDNA, ANCA, anti-U1-nPNP, anti-Sm, anti-centromere and anti-dsDNA antibodies and LAC), and negative anti-*β*2GP1 antibody. Color Doppler ultrasound showed bilateral popliteal vein embolism. SLE ccould involve multiple organs and present corresponding clinical manifestations, commonly including lupus nephritis, blood system involvement, and abundant effusions in serous cavities, which are more common in fertile women. APS is also a non- inflammatory autoimmune disease characterized by recurrent thrombosis, abnormal pregnancy, positive aPL, and tends to affect young women (8). aCL plays a role in the development of the primary NS (17). Previous studies have shown that aPL in serum is closely related to thrombosis in children with SLE and APS. aPL is a group of antibodies that immunoreact with a variety of antigenic substances containing phospholipid structures, including LAC, aCL and anti-*β*2GP1 antibodies, causing thrombus formation by influencing coagulation, anticoagulation and fibrinolysis (6). SLE patients are prone to thrombi due to vasculitis, hypoalbuminemia and other reasons, and the incidence of thrombosis increases significantly after APS is combined (18). Besides, there are rare pediatric cases of clinical SLE coexisting with APS. Notably, the current patient initially presented with NS complicated by arteriovenous thrombosis. In the first course of the disease of the patient, the related antibodies of SLE and APS were all negative. After 5 years of effective treatment, edema and proteinuria appeared again, accompanied by popliteal vein thrombosis. There was no similar case reported before. Clinically, in addition to the three major characteristics of recurrent thrombosis, abnormal pregnancy and positive aPL, there are also some atypical symptoms in APS children, such as cardiovascular disease, migraine, heart valve damage, pulmonary hypertension and livedo reticularis (19), and our patient also presented with livedo reticularis on both lower extremities during the second course of the disease. But the patient developed recurrent arteriovenous thrombosis at the onset, which was not in line with the diagnosis of SLE at that time, and the antiphospholipid antibody was negative, so it was considered to be related to the vascular embolism caused by NS. However, children with recurrent vascular embolism in clinical practice should be considered the possibility of APS. It is recommended to regularly follow up autoantibodies and monitor related immune indicators to detect potential secondary factors early, such as SLE or other immune-related diseases. At the same time of diagnosis of SLE, it is recommended to perform the APS test as a routine follow-up index, at least once a year to recognize APS as early as possible.

The treatment of APS in children is challenging, especially for APS secondary to SLE, which has a worse prognosis and higher mortality compared with adult patients (20). The treatment strategies for childhood APS are based on a small number of observational cohort studies in children and modified adult programs. However, because children have completely different characteristics from adults, long-term preventive anticoagulants are not recommended for asymptomatic aPL-positive children. In the presence of congenital or secondary procoagulant risk factors, multiple aPLs, LA, high titers of ACA and/or anti-*β*2GP1 antibodies, and low molecular weight heparin should be administered prophylactically in high-risk situations (eg. Surgery) (21). The current patient was characterized by lower limb venous thrombosis and right pulmonary artery trunk embolism at the beginning of the disease, with unstable vital signs. After multidisciplinary discussion, lower limb filter implantation and thrombolytic therapy were performed, followed by anticoagulation with low molecular weight heparin, sequential warfarin maintenance therapy, and regular monitoring to maintain INR at 2.0-3.0. In addition, hydroxychloroquine has moderate anticoagulant effects and reduces the release of tissue factors from endothelial cells and leukocytes (22). There is good evidence for its prevention of arterial and venous thrombosis in adults. The current patient was given oral hydroxychloroquine for 18 months after a clear diagnosis, and then gradually reduced and discontinue this medication. Regarding the treatment of the primary disease of SLE coexisting with APS, the patient received aggressive immunosuppressive therapy (high-dose methylprednisolone pulse therapy followed by oral prednisone; cyclophosphamide pulse therapy and then sequential mycophenolate mofetil to suppress immunity), and in the case of poor disease control, CD20 monoclonal antibody (rituximab) and stem cell transplantation were performed. Fortunately, the patient's condition improved significantly, and the drugs were gradually discontinued after 2 years of treatment. Now the patient has been followed up for 5 years, and the disease has not recurred.

## Conclusion

In conclusion, this paper alerts us to the possibility of APS for children with recurrent thrombosis, and regularly monitors antiphospholipid antibodies to avoid missed diagnosis and misdiagnosis and delay of disease. For children with confirmed SLE coexisting with APS, the disease activity index and the severity of organ damage are directly related to a worse prognosis quoad vitam. The higher the activity and the more severe the organ damage, the higher the mortality rate. Therefore, early reasonable treatment, protection of organ function, infection control and regular follow-up could effectively improve the prognosis. Although this patient's condition is complicated, we have always been vigilant about the possibility of APS. During the course of the disease, the antiphospholipid antibody and lupus antibody were tested many times, the diagnosis was made comprehensively, and active and reasonable treatment was conducted, which have significantly improved the prognosis of the patient. Thereafter, regular follow-up should be performed, especially during pregnancy, and more long-term attention should be paid to the presence of thrombotic events in other sites.

## Nomenclature

SLE: Systemic lupus erythematosus, NS: nephrotic syndrome, APS: antiphospholipid syndrome, LAC: lupus anticoagulant, ESR: erythrocyte sedimentation rate, PT: prothrombin time, APTT: activated partial thromboplastin time, CRP: C-reactive protein, CTA: CT angiography, DSA: digital substraction angiography, LN: lupus nephritis, MMF: mycophenolate mofetil.

## Data Availability

The raw data supporting the conclusions of this article will be made available by the authors, without undue reservation.
